# Nutrient Contribution and Acceptability of Dried Small Indigenous Fish Powders for Complementary Feeding in Northern Cameroon

**DOI:** 10.1002/fsn3.72116

**Published:** 2026-07-14

**Authors:** Abomo Ndzana Anne Christine, Essa'a Veronique Josette, Socpa Antoine, Nankap Martin, Medoua Nama Gabriel

**Affiliations:** ^1^ Center for Food and Nutrition Research IMPM Yaoundé Cameroon; ^2^ School of Health Sciences Catholic University of Central Africa Yaoundé Cameroon; ^3^ Department of Anthropology University of Yaoundé 1 Yaoundé Cameroon; ^4^ UNICEF Yaoundé Yaoundé Cameroon

**Keywords:** complementary feeding, fish powder, infant and young child nutrition, nutrient intake, small indigenous fish

## Abstract

Child malnutrition remains a major public health concern in northern Cameroon, where limited access to animal‐source foods contributes to persistent undernutrition and micronutrient deficiencies. This study investigated the nutritional composition of small indigenous fish species, documented household knowledge and consumption practices, and evaluated the acceptability of a small‐fish–based complementary food. A mixed‐methods design was implemented across six localities in the North and Far North regions, combining household surveys (*n* = 639), qualitative interviews with actors in the fish value chain, nutrient analyses of 19 fish samples, and an acceptability test among children aged 6–23 months (*n* = 104). Dried small fish showed high nutrient density, with 50.3–64.1 g protein, 11.2–16.7 g fat, and up to 1412 mg calcium per 100 g. A 10 g portion contributed 40%–50% of daily protein needs and up to one‐third of calcium requirements for young children. Despite widespread household consumption (92.5%), only 24.6% incorporated small fish into children's meals, reflecting gaps in caregiver knowledge of complementary feeding. The standardized fish powder was well accepted, with mean of hedonic scores of 5.8 ± 1.2 for taste, 5.6 ± 1.3 for odor, and 5.7 ± 1.1 for overall appreciation (*p* < 0.05 compared with the neutral midpoint of 4.0). Caregivers reported ease of incorporation into common child foods, indicating strong feasibility for household use. These findings demonstrate that small indigenous fish are nutrient‐dense, culturally acceptable, and feasible to integrate into complementary feeding. Given the observational nature of the study, conclusions remain cautious. Future research should assess long‐term nutritional impacts, evaluate product stability and safety under household storage, and explore scalable models for community‐based production.

## Graphical abstract

This study investigated the nutritional composition, household practices, and acceptability of small indigenous fish for complementary feeding in northern Cameroon. A mixed‐methods design included household surveys (*n* = 639), nutrient analyses of 19 dried fish samples, and an acceptability test among children aged 6–23 months (*n* = 104) across six localities. hile 92.5% of households reported consuming small fish, only 24.6% of caregivers incorporated them into child meals, revealing a gap in complementary feeding practices. Dried small fish were highly nutrient‐dense, containing 50–64 g protein, 11–17 g fat, and 1412 mg calcium per 100 g. A 10 g portion provided 40%–50% of daily protein requirements and approximately one‐third of calcium needs for young children. Fish powder prepared from small fish was well accepted, with mean hedonic scores of 5.8 ± 1.2 for taste, 5.6 ± 1.3 for odor, and 5.7 ± 1.1 overall (*p* < 0.05). Small indigenous fish are nutrient‐dense and culturally acceptable, representing a feasible option for improving complementary feeding. Further research is required to assess long‐term nutritional impacts and ensure product safety.

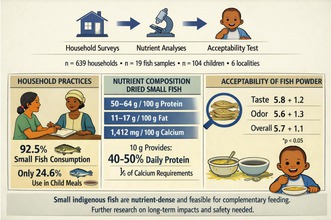

## 1 Introduction

Child malnutrition continues to pose a major challenge in Cameroon's North and Far North regions, where chronic food insecurity, poverty, and restricted access to animal‐source foods undermine dietary quality. More than 40% of children under five are stunted, and deficiencies in iron, zinc, and vitamin A remain widespread (Institut National de la Statistique (INS) and ICF 2020; Ministry of Public Health 2009). Identifying locally available, nutrient‐rich foods that can be feasibly integrated into complementary feeding is therefore a public health priority.

Aquatic foods have recently gained attention as part of the global policy frameworks, including the FAO High Level Panel of Experts (HLPE 2014; HLPE 2021). These frameworks emphasize the role of aquatic foods as affordable, culturally embedded sources of highly bioavailable micronutrients. Research from Asia and West Africa shows that small fish are an important source of calcium, iron, zinc, vitamin A, vitamin D, and essential fatty acids (Kawarazuka and Béné 2011; Thilsted et al. 2016; Bogard et al. 2015). However, despite this global recognition, scientific evidence on the nutrient composition, bioaccessibility, and acceptability of small indigenous fish in Central Africa remains scarce.

Cameroon represents a unique context for such investigation. Small fish species such as *Soudamouka* and *Gamré* are widely consumed by adults and older children, yet their integration into infant and young child diet is minimal, despite local abundance. This limited use may be attributed either to gaps in caregiver knowledge regarding complementary feeding practices or to the absence of systematic evaluation of small fish as a targeted nutrition strategy.

Fish powder specifically warrants investigation because it offers a practical, safe, and culturally adaptable form for incorporation into children's meals. Recent studies have demonstrated that fish powders can improve nutrient intake when integrated into complementary foods, particularly by enhancing calcium, iron, and zinc density in porridges and weaning diets (Byrd et al. 2021; Thilsted et al. 2016). Evidence from fortification trials in Asia and Africa shows that fish powder can be successfully blended into cereal‐based foods without compromising sensory acceptability (Roos et al. 2007). Moreover, emerging work on nutrient bioaccessibility highlights that minerals from small fish are highly bioavailable compared to plant‐based sources, reinforcing their potential role in addressing micronutrient deficiencies (Roos et al. 2007). Despite these advances, data on nutrient composition, bioaccessibility, and acceptability of small indigenous fish powders in Cameroon remain scarce. Addressing these limitations is essential to determine the potential of locally available species such as *Gamré* and *Soudamouka* to serve as a food‐based strategy for improving child nutrition in this setting.

This study therefore (i) characterizes the nutritional properties of small fish commonly consumed in northern Cameroon, (ii) documents household knowledge, consumption patterns, and perceptions, and (iii) evaluates the acceptability of a standardized preparation of small fish powder intended for infant and young child feeding. By linking nutrient composition with cultural practices and caregiver perceptions, the study aims to establish whether small indigenous fish can serve as a feasible and nutritionally effective approach to strengthen complementary feeding in this region.

## 2 Materials and Methods

### 2.1 Study Design and Rationale

A mixed‐methods descriptive and analytical design was adopted to capture both the quantitative prevalence of household practices and the qualitative depth of community perceptions regarding small indigenous fish. This approach was chosen to ensure triangulation of findings: quantitative surveys provided measurable indicators of consumption and feeding practices, while qualitative interviews and focus groups contextualized these behaviors within cultural norms and value chains. Laboratory analyses complemented field data by objectively characterizing nutrient composition. Together, these components allowed for a comprehensive assessment of feasibility and acceptability.

### 2.2 Study Setting, Sampling Strategy, and Data Collection

The study was conducted between July and September 2024 in six purposively selected localities of the North and Far North regions of Cameroon (Garoua, Lagdo, Maroua, Pouss, Magda, and Mokolo). Selection criteria included proximity to fishing areas, accessibility, and relevance to local dietary practices. Exclusion criteria included localities with ongoing humanitarian interventions that could bias dietary practices, areas with security concerns or logistical barriers preventing safe data collection, and communities where fish consumption was negligible or culturally restricted. The study combined quantitative household surveys, qualitative interviews with actors in the fish value chain, and laboratory analyses of small fish samples.

#### 2.2.1 Household Survey

A cross‐sectional design targeted households with at least one child under 5 years. A sample size of 639 households was achieved using stratified random sampling within each locality to ensure representation across socioeconomic strata.

Structured questionnaires were administered by trained field workers in local languages. Data captured sociodemographic characteristics, child feeding practices, dietary diversity, knowledge and consumption of small fish, and willingness to incorporate them into complementary foods.

To ensure validity and reliability, the questionnaire was pretested with 30 households in a comparable locality not included in the main study. Feedback from this pilot was systematically used to refine item wording, sequencing, and clarity. The internal consistency of multi‐item constructs (e.g., caregiver perceptions of acceptability) was assessed using Cronbach's alpha, with coefficients ≥ 0.70 considered indicative of satisfactory reliability. In addition, content validity was established through expert review by specialists experienced in complementary feeding practices in Cameroon, ensuring that the instrument adequately captured the intended domains.

#### 2.2.2 Qualitative Interviews and Focus Group Discussions

Participants (fishers, processors, traders, and caregivers) were recruited through purposive sampling to capture diverse perspectives along the fish value chain.

Semi‐structured interviews were conducted with 42 participants across the six study sites, complemented by six focus group discussions (8–10 participants each). Discussions explored fishing practices, seasonal availability, processing methods, and perceptions of small fish in child diets. Interviews and discussions continued until thematic saturation was reached, that is, no new themes emerged. Transcripts were coded inductively and analyzed using thematic content analysis. Coding was performed independently by two researchers, with discrepancies resolved through consensus. NVivo software was used to manage data and ensure transparency of coding procedures.

#### 2.2.3 Fish Ample Collection

Fresh and dried samples of seven fish types were collected for analysis. In addition to local names, species were identified by their Latin binomials: *Gamré* (*Chelaethiops soudaensis*), *Soudamouka* ( *Petrocephalus soudanensis* ), Juvenile Carp ( *Labeo coubie* ), Juvenile freshwater sardine ( *Alestes dentex* ), *Kétchopérado* ( *Alestes baremoze* ), *Souda'odjé* ( *Chelaethiops bibie* ), and *Pelpéladji* (*Enteromius baudoni*). Samples were obtained from local markets or directly from fishing sites, selected for freshness, representation of commonly consumed species, and diversity of processing methods. Fresh fish were transported under cold‐chain conditions (−40°C to −20°C) and stored at −21°C until analysis, while dried samples were kept in insulated containers at 0°C–4°C to maintain quality before laboratory assessment.

#### 2.2.4 Laboratory Analyses

Nutrient composition was determined on 10 g of dry matter per sample using established analytical methods. Dry matter content was measured by oven drying at 105°C (AOAC 927.05). Protein was quantified by the Kjeldahl method, using a nitrogen‐to‐protein conversion factor of 6.25 (AOAC International 1990). Lipid content was assessed by Soxhlet extraction with petroleum ether (AOAC International 1990). Carbohydrates were determined using the Fischer and Stein (1961) method, and ash content was measured by incineration at 550°C (AOAC 920.87). Mineral concentrations (Ca, Fe, Zn) were analyzed by atomic absorption spectrophotometry, while vitamin A was quantified following AACC Method 86‐05.01 (AACC International 2010). Metabolizable energy was calculated using FAO conversion factors (Food and Agriculture Organization of the United Nations 2003). In all samples, values below the detection limit were reported as 0.00 g/100 g.

The contribution of a 10 g portion of dried small fish to the Recommended Daily Intakes for children aged 6–21 months was estimated using WHO/FAO (2004), Dewey (2003), and Institute of Medicine (2001, 2005) reference values. These contributions were performed as theoretical maximum estimates. They assume complete intake, high bioavailability, and no processing losses, and are therefore intended to illustrate potential nutritional significance rather than actual realized intake and should therefore be interpreted as indicative upper‐bound potentials, not precise estimates of effective nutrient delivery under household conditions.

### 2.3 Processing of Small Fish and Acceptability Assessment

#### 2.3.1 Processing of Small Fish

Fresh *Gamré* fish were thoroughly cleaned to remove surface impurities and cooked under controlled conditions to ensure microbial inactivation (FAO/WHO 2009). The cooked fish were then dried in a hygienic environment using standardized protocols to achieve a stable moisture content (< 10%), thereby reducing risks of microbial growth and lipid oxidation (Sikorski and Kolakowska 2002). The dried material was subsequently milled into a fine powder using sanitized equipment, and particle size was standardized to ensure uniformity and product consistency. The final powder was packaged in airtight, food‐grade containers to limit oxygen exposure and prevent cross‐contamination, and stored at ambient temperature in a clean, dry environment.

Before household testing, the fish powder was assessed for homogeneity, moisture content, microbial safety (total plate count and coliform absence), and sensory stability. Standardization procedures ensured that all samples used in the acceptability assessment were identical in composition, texture, and appearance. Together, these measures reduced the risks of microbial contamination, pathogenic organisms, and oxidative spoilage, thereby ensuring the product's safety for infants (Mortimore and Wallace 2013; Wild and Gong 2010).

#### 2.3.2 Acceptability Assessment

Acceptability testing was conducted among 104 children aged 6–23 months. During each feeding session, caregivers were instructed to mix one spoonful of fish powder (approximately 10 g) into the child's prepared meal and feed the child under direct observation. Because children in this age group cannot reliably perform hedonic evaluations, caregivers provided ratings on a 0–7 hedonic scale for taste, odor, texture, appearance, and overall appreciation, based on their direct observations of child reactions (acceptance, refusal, facial expressions, and feeding ease). Trained field workers documented caregiver assessment and child responses using standardized observation forms to ensure consistency and reliability of data collection.

### 2.4 Statistical Analyses

Quantitative survey data were entered into SPSS v25 and analyzed using descriptive statistics (frequencies, means, and proportions). The nutrient contributions of 10 g portions of dried small fish were compared against WHO/FAO recommended intakes for children aged 6–21 months.

To identify independent determinants of small fish utilization in children's meals, multivariate logistic regression analyses were performed. Predictor variables included caregiver education, household size, religion, residence, and household head occupation. Adjusted odds ratios (AOR) with 95% confidence intervals (CI) were reported, and statistical significance was set at *p* < 0.05.

Qualitative data from interviews and focus groups were transcribed verbatim and coded inductively in NVivo v12. A thematic analysis was applied to identify cultural perceptions, barriers, and facilitators of small fish use in child feeding. Coding reliability was ensured through double coding and consensus resolution among researchers.

Laboratory data were expressed as mean ± standard deviation (SD). Comparisons across species and between fresh and dried forms were performed using Duncan's multiple range test. Statistical significance was set at *p* < 0.05.

Finally, findings from quantitative, qualitative, and laboratory strands were triangulated. Convergence and divergence across datasets were explicitly examined to strengthen validity and provide a nuanced interpretation of nutritional potential, household practices, and product acceptability.

## 3 Results

### 3.1 Sociodemographic Characteristics

Characteristics of the surveyed households are summarized in Table 1. The study included 639 households, with respondents predominantly female (90.9%), reflecting the central role of women in childcare and household nutrition. The mean respondent age was 30 ± 9 years. Most respondents were wives (63.9%) or mothers (23.3%), indicating that the survey largely captured the perspectives of primary caregivers. Educational attainment was modest: 40.6% had completed primary school, 28.1% had lower secondary education, 12.7% had attained higher education, and 16.4% reported Koranic schooling.

**TABLE 1:** Sociodemographic characteristics of surveyed households.

Variable	Category	*n* (%)
Respondent's sex	Female	581 (90.9)
	Male	58 (9.1)
Respondent's age (years)	Mean ± SD	30 ± 9 (*n* = 639)
Marital status	Single	41 (6.4)
	Divorced	17 (2.7)
	Monogamous union	356 (55.6)
	Polygamous union	197 (30.8)
	Widowed	26 (4.1)
Relationship to household head	Wife	409 (63.9)
	Daughter	13 (2.0)
	Mother	149 (23.3)
	Sister	32 (5.0)
	Other	33 (5.2)
Education level	Literate in local culture	13 (2.0)
	Koranic	105 (16.4)
	Primary	260 (40.6)
	Lower secondary	180 (28.1)
	Higher education	81 (12.7)
Household status	Internally displaced	50 (7.8)
	Refugee	6 (0.9)
	Resident	572 (89.4)
Ethnic group (household head)	Fulbe	341 (53.3)
	Giziga	31 (4.8)
	Mundang	42 (6.6)
	Tupuri	42 (6.6)
	Zumaya	1 (0.2)
	Other	182 (28.4)
Religion	Buddhist	2 (0.3)
	Catholic	93 (14.5)
	Muslim	452 (70.6)
	Protestant	83 (13.0)
	Other	9 (1.5)
Occupation (household head)	Farmer	136 (21.3)
	Livestock breeder	23 (3.6)
	Large‐scale trader	18 (2.8)
	Laborer	33 (5.2)
	Fisher	144 (22.5)
	Small‐scale trader	197 (30.8)
	Other	87 (13.6)
Household size	≤ 5 members	326 (59.8)
	> 5 members	219 (40.2)
Children < 5 years	1–2 children	549 (87.8)
	≥ 3 children	76 (12.2)
Child's sex	Female	260 (42.9)
	Male	346 (57.1)
Child's age (months)	0–5	64 (10.3)
	6–11	85 (13.6)
	12–23	159 (25.5)
	24–35	154 (24.7)
	36–47	88 (14.1)
	48–59	73 (11.7)

Household structures were mainly monogamous (55.6%), although polygamous households represented a substantial proportion (30.8%). The majority of households were long‐term residents (89.4%), with smaller proportions of internally displaced persons (7.8%), and refugees (0.9%). More than half of households (59.8%) had five or fewer members, and most had one to two children under 5 years of age (87.8%).

The ethnic composition was dominated by the Fulbe group (53.3%), followed by minority groups such as Mundang, Tupuri, and Giziga. Islam was the predominant religion (70.6%), with Catholics (14.5%) and Protestants (13.0%) forming notable minorities. Household heads were primarily engaged in small‐scale trading (30.8%), fishing (22.5%), or farming (21.3%), reflecting a largely informal, livelihood‐based economy.

Among children under 5 years, 57.1% were male and 42.9% female. Most children were between 12 and 35 months of age, corresponding to the critical complementary feeding period.

These characteristics contextualize household nutrition practices, highlighting the socioeconomic constraints within which complementary feeding decisions are made.

### 3.2 Child Feeding Practices

Table 2 presents the dietary intake of children under five in the 24 h preceding the survey. Food consumption patterns showed considerable variability in breastfeeding status, dietary diversity, and complementary feeding practices. Slightly more than half of the children were no longer breastfed (53.1%), while 46.9% continued to receive breast milk. Dietary diversity was generally favorable, with 51.7% of children consuming six or more food groups; however, 28.7% had low diversity (≤ 3 food groups), suggesting that a substantial proportion may be at risk of inadequate micronutrient intake. Meal frequency was adequate for most children: 60.3% consumed more than three meals per day and 37.5% consumed three meals, whereas only 2.2% received fewer than three meals daily.

**TABLE 2:** Food consumption among children under 5 years.

Variable	Categories	*n* (%)
Breastfed	No	339 (53.1)
	Yes	299 (46.9)
Dietary diversity	Low (≤ 3 food groups)	175 (28.7)
	Medium (4–5 food groups)	119 (19.5)
	High (≥ 6 food groups)	315 (51.7)
Number of meals per day	< 3 meals	12 (2.2)
	3 meals	203 (37.5)
	> 3 meals	327 (60.3)
Foods used to enrich porridge	Animal‐source foods	120 (18.7)
	Vitamin A‐rich fruits and vegetables	51 (7.9)
	Oils, butter	157 (24.5)
	Dark green leafy vegetables	28 (4.4)
	Legumes, nuts, and seeds	135 (21.0)
	Don't know	151 (23.5)

The types of foods used to enrich porridge varied widely. Oils and butter were the most frequently used enrichers (24.5%), followed by legumes, nuts, and seeds (21.0%) and animal‐source foods (18.7%). In contrast, the use of vitamin A–rich fruits and vegetables (7.9%) and dark green leafy vegetables (4.4%) was limited, indicating suboptimal incorporation of micronutrient‐dense ingredients. Notably, 23.5% of caregivers reported not knowing which foods were appropriate for enrichment, highlighting persistent gaps in nutrition knowledge. Overall, while many children achieved adequate dietary diversity and meal frequency, the limited use of nutrient‐rich foods and the high proportion of caregivers lacking guidance underscore the need for strengthened nutrition education to improve complementary feeding practices.

These findings suggest that although meal frequency was generally adequate, the quality of complementary foods remains suboptimal, with limited incorporation of nutrient‐dense ingredients.

### 3.3 Knowledge and Consumption of Small Fish

Findings related to household knowledge and consumption of small fish are summarized in Table 3. Nearly all respondents (89.4%) reported knowing small fish, with several local names cited—*Gambré* (37.5%) and *Soudamouka* (32.4%) being the most common. Consumption was similarly high, with 92.5% of households reporting that they consumed small fish. The primary reasons for consumption included good taste (54.6%), perceived nutritional value (24.0%), ease of consumption (12.4%), and contribution to dietary diversification (9.0%). Most households consumed small fish once per week (60.0%), while 30.0% consumed them twice weekly and 10.0% more than twice.

**TABLE 3:** Knowledge and consumption of small fish among households.

Variable	Category	*n* (%)
Knowledge of small fish	No	68 (10.6)
	Yes	571 (89.4)
Local names (native language)	Gambré	214 (37.5)
	Soudamouka	185 (32.4)
	Souddodji	23 (4.0)
	Other^a^	149 (26.1)
Consumption of small fish	No	47 (7.5)
	Yes	583 (92.5)
Reasons for consuming small fish	Good taste	316 (54.6)
	Nutritious	139 (24.0)
	Easy and quick to eat	72 (12.4)
	Helps diversify the diet	52 (9.0)
Weekly consumption frequency	Once	346 (60.0)
	Twice	173 (30.0)
	More than twice	58 (10.0)
Child consumes small fish	No	181 (28.2)
	Yes	441 (68.7)
Use of small fish to enrich child meals	No	484 (75.4)
	Yes^b^	158 (24.6)
Source of supply	Markets	440 (76.1)
	Fishing areas	98 (17.0)
	Other	40 (6.9)
Form of use	Fresh	287 (50.7)
	Dried	279 (49.3)
Perception of smell	Pleasant	344 (59.5)
	Acceptable	102 (17.7)
	Bad	132 (22.8)
Willingness to include small fish in child's diet	Favorable	525 (91.8)
	Not favorable	47 (8.2)
Weekly expenditure on small fish (FCFA)	500–1500	331 (57.7)
	1500–3000	195 (34.0)
	> 3000	48 (8.3)

^a^ Forty‐eight other local names mentioned by 1–4 respondents.

^b^ Mainly used to enrich porridges, sauces, and vegetables.

Children's consumption of small fish was also common (68.7%); however, only 24.6% of households used small fish to enrich children's meals, despite their nutrient density. Markets were the predominant source of supply (76.1%), followed by fishing areas (17.0%). Small fish were used in both fresh (50.7%) and dried (49.3%) forms. Perceptions of smell were generally positive or acceptable (77.2%), although 22.8% considered the smell unpleasant. Importantly, willingness to include small fish in children's diets was very high (91.8%), indicating strong acceptability. Weekly expenditures were modest, with most households spending 500–1500 FCFA (57.7%) or 1500–3000 FCFA (34.0%). Overall, the findings highlight high awareness, consumption, and acceptability of small fish, but also reveal a missed opportunity to promote their use in complementary feeding.

**FIGURE 1:**
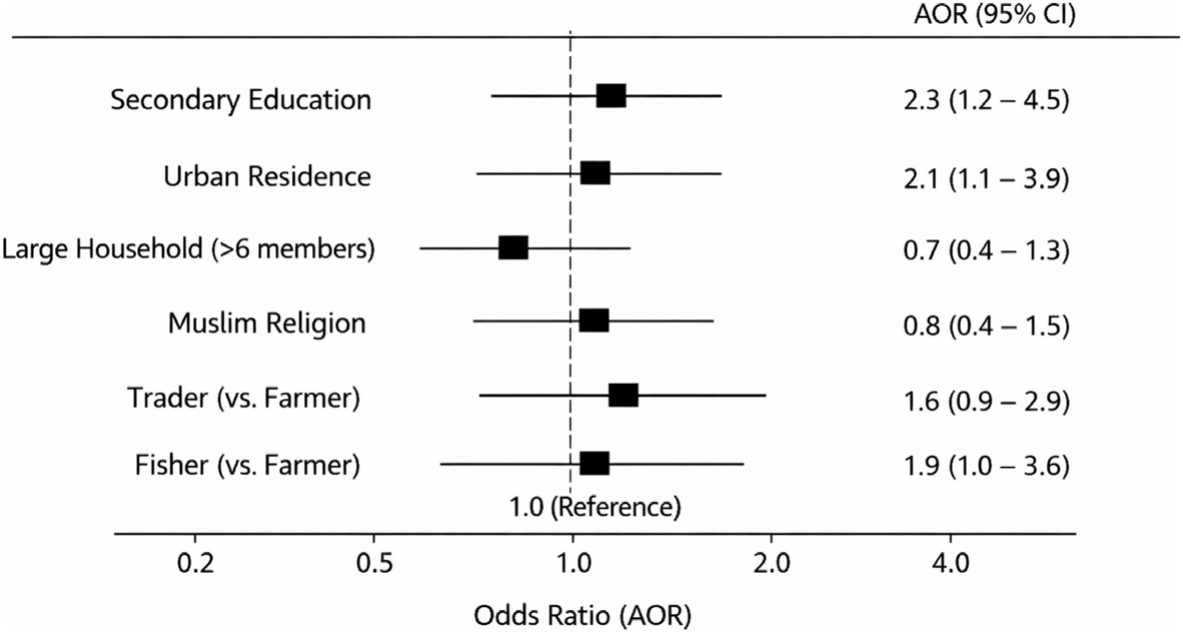
Forest plot of adjusted odds ratios (AOR) for determinants of small fish utilization in children's meals. Squares represent adjusted odds ratios, and horizontal lines indicate 95% confidence intervals derived from multivariate logistic regression. The vertical dashed line at 1.0 marks the reference value. Caregiver education, urban residence, and household head occupation (trading/fishing) were associated with a higher likelihood of small fish incorporation (*p* < 0.05), whereas household size and religion were not significant predictors.

Multivariate analysis, presented in Table 4 and Figure 1, confirmed that caregiver education, urban residence, and household head occupation were significant determinants of small fish utilization. Caregivers with secondary or higher education (AOR = 2.3, 95% CI: 1.2–4.5) and urban households (AOR = 2.1, 95% CI: 1.1–3.9) were more likely to incorporate small fish. Households headed by traders (AOR = 1.6, 95% CI: 0.9–2.9) and fishers (AOR = 1.9, 95% CI: 1.0–3.6) also showed higher utilization compared to farmers. Household size and religion were not significant predictors.

**TABLE 4:** Multivariate logistic regression of determinants of small fish utilization in children's meals.

Predictor	Adjusted odds ratio (AOR)	95% CI	*p*
Caregiver education			
Secondary or higher vs. primary/none	2.3	1.2–4.5	0.01
Residence			
Urban vs. rural	2.1	1.1–3.9	0.02
Household size			
> 6 members vs. ≤ 6 members	0.7	0.4–1.3	0.18
Religion			
Muslim vs. Christian	0.8	0.4 − 1.5	0.27
Household head occupation			
Trader vs. farmer	1.6	0.9–2.9	0.08
Fisher vs. farmer	1.9	1.0–3.6	0.04

### 3.4 Nutritional Composition of Small Fish Species

Nutrient composition results are presented in Table 5. The analyzed small fish species showed substantial variation in nutritional content across species and between fresh and dried forms. As expected, dried samples exhibited markedly higher energy densities (346.8–401.5 kcal/100 g) compared with fresh fish (123.9–174.8 kcal/100 g), reflecting the sharp reduction in moisture content following drying. Protein concentrations were also considerably higher in dried samples (50.3–64.1 g/100 g) than in fresh samples (20.3–26.2 g/100 g), consistent with nutrient concentration due to water loss. Fat content followed a similar pattern, with dried fish containing 11.2–16.7 g/100 g compared with 4.3–7.8 g/100 g in fresh samples. Carbohydrate values were consistently negligible across all species, reflecting the minimal glycogen reserves in fish muscle, which are rapidly depleted post‐mortem (Love 1970; Huss 1995).

**TABLE 5:** Nutritional composition of small fish samples.

Sample	Energy (kcal/100 g)	Moisture (g/100 g)	Protein (g/100 g)	Fat (g/100 g)	Carbohydrates (g/100 g)	Ash (g/100 g)	Calcium (mg/100 g)	Iron (mg/100 g)	Zinc (mg/100 g)	Vitamin A (μg/100 g)
Gamré fresh	131.25 ± 48.87^a^	67.25 ± 10.12^g^	23.10 ± 6.78^a^	4.30 ± 2.40^a^	0.00 ± 0.00^a^	4.95 ± 0.64^a^	342.00 ± 43.22^a^	2.85 ± 0.35^a^	1.85 ± 0.21^a^	10.05 ± 5.59^a^
Gamré dried	375.07 ± 8.13^f^	11.90 ± 0.66^c^	62.10 ± 1.73^f^	14.07 ± 1.72^e^	0.00 ± 0.00^a^	11.47 ± 0.80^d^	1115.93 ± 79.06^j^	6.67 ± 0.50^d^	4.27 ± 0.31^d^	32.80 ± 3.74^d^
Soudamoka fresh	174.8 ± 2.6^b^	61.3 ± 1.0^f^	26.2 ± 0.6^b^	7.8 ± 0.5^b^	0.0 ± 0.0^a^	4.3 ± 0.6^a^	295.2 ± 20.7^a^	2.5 ± 0.3^a^	1.6 ± 0.2^a^	18.1 ± 0.1^b^
Soudamoka dried	379.93 ± 27.41^f^	13.13 ± 4.39^c^	61.58 ± 1.82^f^	14.85 ± 2.28^e^	0.00 ± 0.00^a^	10.03 ± 1.40^c^	840.58 ± 31.72^g^	5.78 ± 0.83^c^	3.73 ± 0.52^c^	34.68 ± 5.15^d^
Juvenile carp fresh	123.9 ± 3.4^a^	72.2 ± 0.4^h^	20.3 ± 0.8^a^	4.7 ± 0.1^a^	0.0 ± 0.0^a^	2.4 ± 0.3^a^	69.6 ± 0.5^a^	1.4 ± 0.2^a^	0.9 ± 0.1^a^	4.1 ± 0.1^a^
Juvenile carp dried	353.2 ± 10.6^e^	13.5 ± 0.4^c^	56.4 ± 4.7^e^	14.2 ± 2.1^e^	0.0 ± 0.0^a^	15.4 ± 3.8^e^	459.6 ± 56.1^d^	8.9 ± 2.2^e^	5.7 ± 1.4^e^	12.3 ± 1.8^a^
Juvenile freshwater sardine fresh	170.8 ± 1.8^b^	62.3 ± 1.4^f^	26.1 ± 0.5^b^	7.4 ± 0.3^b^	0.0 ± 0.0^a^	3.9 ± 0.9^a^	410.0 ± 7.6^c^	2.1 ± 0.1^a^	1.3 ± 0.1^a^	28.0 ± 1.0^c^
Juvenile freshwater sardine dried	357.20 ± 13.15^e^	11.35 ± 2.33^b^	64.05 ± 0.07^g^	11.20 ± 1.41^d^	0.00 ± 0.00^a^	13.00 ± 0.71^d^	1412.30 ± 77.85^k^	7.50 ± 0.42^d^	4.80 ± 0.28^d^	42.60 ± 5.38^e^
Kétchopérado dried	401.5 ± 4.2^g^	9.8 ± 0.3^b^	62.8 ± 0.2^f^	16.7 ± 0.4^f^	0.0 ± 0.0^a^	10.1 ± 0.2^c^	698.6 ± 6.8^f^	5.8 ± 0.1^c^	3.7 ± 0.1^c^	38.9 ± 1.0^d^
Souda'odjé dried	377.6 ± 0.8^f^	7.4 ± 0.3^a^	63.1 ± 0.4^g^	13.9 ± 0.2^e^	0.0 ± 0.0^a^	15.1 ± 0.2^e^	1048.8 ± 7.5^i^	8.8 ± 0.1^e^	5.6 ± 0.1^e^	32.4 ± 0.4^d^
Pelpéladji dried	346.8 ± 10.1^d^	20.0 ± 0.5^d^	50.3 ± 3.7^d^	16.2 ± 1.0^f^	0.0 ± 0.0^a^	12.9 ± 2.4^d^	898.8 ± 82.0^h^	7.5 ± 1.4^d^	4.8 ± 0.9^d^	37.7 ± 2.3^d^

*Note:* Values are expressed as mean ± SD (*n* = 3). Superscript letters indicate groups not significantly different within columns according to Duncan's test.

Mineral content increased substantially with drying. Calcium levels were particularly elevated in dried *Sardine* (1412.3 mg/100 g), *Gamré* (1115.9 mg/100 g), and *Souda'odjé* (1048.8 mg/100 g), whereas fresh samples contained considerably lower amounts. Iron and zinc concentrations were also higher in dried fish, with dried Carp showing the highest iron (8.9 mg/100 g) and zinc (5.7 mg/100 g) contents. Vitamin A levels varied across species but were consistently higher in dried samples, with dried *Sardine* (42.6 μg/100 g), *Kétchopérado* (38.9 μg/100 g), and *Pelpéladji* (37.7 μg/100 g) exhibiting the greatest concentrations.

Overall, drying significantly enhanced the nutrient density of all fish species, particularly for protein, fat, minerals, and vitamin A. These findings underscore the value of dried small fish as concentrated sources of essential nutrients and highlight their potential contribution to improving dietary quality in settings where micronutrient deficiencies are prevalent.

#### 3.4.1 Nutrient Content Per 10 g of Dried Small Fish

Table 6 compares the nutrient contribution of a 10 g portion of dried small fish with the recommended daily intakes (RDA) for children aged 6–21 months. The results highlight the exceptional nutrient density of dried small fish and their potential to address key micronutrient gaps in complementary feeding. Calcium contributions were particularly notable: dried *Sardine* (141.2 mg) and dried *Gamré* (111.6 mg) provided the highest amounts, corresponding to approximately one quarter to one third of daily requirements. Dried *Soudamoka* (84.1 mg) and dried *Souda'odjé* (104.9 mg) also contributed meaningful quantities, while even the lowest‐calcium species, dried Carp (45.9 mg), supplied around 10% of daily needs.

**TABLE 6:** Contribution of 10 g of dried small fish to recommended daily intakes for children 6–21 months.

Nutrient	Calcium (mg)	Iron (mg)	Zinc (mg)	Vitamin A (μg)	Protein (g)	Fat (g)
RDA for children 6–21 months^a^	400–500 mg/day	11 mg/day	3 mg/day	400 μg/day	~13–14 g/day from complementary foods	No fixed RDA; important for energy density
Gamré dried (per 10 g)	111.6	0.67	0.43	3.28	6.21	1.41
Soudamoka dried (per 10 g)	84.1	0.58	0.37	3.47	6.16	1.49
Juvenile carp dried (per 10 g)	45.9	0.89	0.57	1.23	5.64	1.42
Juvenile freshwater sardine dried (per 10 g)	141.2	0.75	0.48	4.26	6.41	1.12
Kétchopérado dried (per 10 g)	69.9	0.58	0.37	3.89	6.28	1.67
Souda'odjé dried (per 10 g)	104.9	0.88	0.56	3.24	6.31	1.39
Pelpéladji dried (per 10 g)	89.9	0.75	0.48	3.77	5.03	1.62

*Note:* Values represent theoretical maximum contributions to recommended dietary allowances (RDA). Calculations assume complete intake, high bioavailability, and no processing losses. They should therefore be interpreted as indicative upper‐bound potentials rather than actual realized intakes under household conditions.

^a^ RDA values reflect WHO/FAO and IOM recommendations for complementary feeding (6–23 months), adapted for the 6–21‐month window.

Iron contributions, although modest relative to the high requirement of 11 mg/day, were nutritionally relevant. Dried Carp (0.89 mg) and dried *Souda'odjé* (0.88 mg) provided the highest amounts, followed by dried *Sardine* (0.75 mg) and dried *Gamré* (0.67 mg). Zinc contributions were more substantial in relative terms: dried Carp (0.57 mg) and dried *Souda'odjé* (0.56 mg) covered nearly one fifth of the daily requirement, while dried *Gamré* (0.43 mg) and dried *Sardine* (0.48 mg) also contributed significantly.

Vitamin A contributions were modest across species, with dried *Sardine* (4.26 μg) and dried *Kétchopérado* (3.89 μg) providing the highest amounts, though still representing only about 1% of daily needs. In contrast, protein contributions were substantial: all species provided between 5.0 and 6.4 g of protein per 10 g portion, representing approximately 40%–50% of the daily protein requirement from complementary foods. Fat contributions ranged from 1.1 to 1.7 g per 10 g, supporting the energy density required in complementary feeding.

Overall, the findings demonstrate that even small quantities of dried small fish can make meaningful contributions to the micronutrient and protein needs of young children. Dried *Gamré* and dried *Sardine* stand out for their high calcium density, while dried Carp and dried *Souda'odjé* provide the greatest contributions to iron and zinc requirements. These results underscore the potential of dried small fish as affordable, nutrient‐dense ingredients for improving complementary feeding practices.

### 3.5 Acceptability Assessment

Table 7 presents the demographic and nutritional characteristics of the 104 children included in the acceptability assessment, reflecting a profile typical of a nutritionally vulnerable population. Girls represented a larger proportion of the sample (60.6%), and the mean age was 14.3 months (range: 6–23 months), corresponding to a period of heightened sensitivity to dietary quality during complementary feeding. Anthropometric measurements showed considerable variability, with mean weight and height of 8.1 kg and 74.6 cm, respectively. The distribution of Z‐scores indicates a substantial burden of undernutrition. The mean weight‐for‐height Z‐score (−1.5) reflects a high prevalence of acute malnutrition, affecting 33.7% of children. Similarly, 34.6% were underweight, consistent with the mean weight‐for‐age Z‐score of −1.5. Chronic malnutrition was also present, with 14.4% of children classified as stunted based on a mean height‐for‐age Z‐score of −0.8. No cases of overweight were observed. Taken together, these indicators highlight a population experiencing significant nutritional stress, with both acute and chronic forms of malnutrition represented. This context underscores the relevance of evaluating nutrient‐dense complementary foods, such as small fish–based products, in a group likely to benefit substantially from improved dietary quality.

**TABLE 7:** Characteristics of children in the acceptability test.

Characteristics	*n* (%) or Mean ± SD	Minimum	Maximum
Sex—Girls	63 (60.6)	—	—
Sex—Boys	41 (39.4)	—	—
Age (months)	14.3 ± 5.2	6	23
Weight (kg)	8.1 ± 1.3	5	12
Height (cm)	74.6 ± 5.5	61.2	88.5
Weight‐for‐Height Z‐score	–1.5 ± 1.2	−4.8	1.8
Acute malnutrition	35 (33.7)	—	—
Height‐for‐Age Z‐score	−0.8 ± 1.3	−4.5	3.9
Chronic malnutrition	15 (14.4)	—	—
Weight‐for‐Age Z‐score	−1.5 ± 1.1	−4.4	1.2
Underweight	36 (34.6)	—	—
Overweight	0 (0)	—	—

**FIGURE 2:**
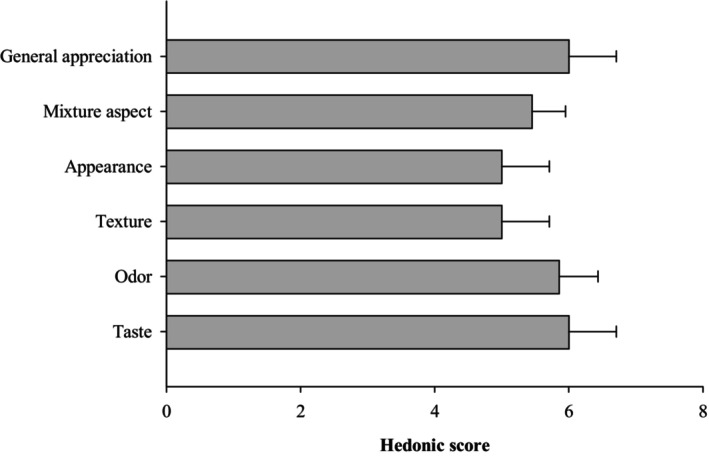
Sensory evaluation scores of the standardized small‐fish–based product obtained during household acceptability testing. Ratings were collected using a 7‐point hedonic scale (0 = “dislike extremely” to 7 = “like extremely”) across six attributes: General appreciation, odor, taste, texture, appearance, and aspect of the mixture. Higher scores indicate greater acceptability of the product among participating households.

Figure 2 displays the average hedonic ratings for six sensory attributes (general appreciation, odor, taste, texture, appearance, and mixture aspect) of the standardized small fish powder. Mean scores ranged from 5.00 to 6.00 on the 7‐point hedonic scale, indicating overall favorable acceptance. General appreciation and taste achieved the highest ratings (mean = 6.00, 95% CI: 5.86–6.14), followed by odor (mean = 5.86, 95% CI: 5.74–5.97). Mixture aspect received intermediate scores (mean = 5.45, 95% CI: 5.35–5.55), while appearance and texture were comparatively lower (mean = 5.00, 95% CI: 4.86–5.14). ANOVA revealed significant differences among attributes (*F* (5,618) = 53.27, *p* < 0.001), confirming that panelists discriminated between sensory dimensions. Post hoc Duncan grouping established a clear hierarchy: appearance and texture formed the lowest subset, mixture aspect was intermediate, odor was higher, and taste together with general appreciation clustered at the top. Taken together, the product demonstrated robust sensory acceptability, with taste and general appreciation as primary strengths, odor contributing positively, and appearance and texture identified as areas requiring improvement.

### 3.6 Analytical Insight

The results reveal a paradox: small fish are widely known, consumed, and culturally accepted, yet their incorporation into children's diets remains limited. This gap underscores a missed opportunity to harness locally available, nutrient‐dense foods to address child malnutrition. The constraint lies not in availability or acceptability, but in the translation of knowledge into practice.

Caregivers demonstrate high awareness and willingness, but limited guidance on preparation, portioning, and safe integration into complementary feeding constrains consistent use. This knowledge–practice disconnect highlights the need for targeted nutrition education, demonstration of preparation techniques, and culturally tailored behavior‐change communication. Such interventions are critical to transform high awareness into sustained, child‐focused utilization of small indigenous fish.

## 4 Discussion

This study provides new evidence on the nutritional value, household perceptions, and acceptability of small indigenous fish as complementary food ingredients in northern Cameroon. The findings highlight the potential of species such as *Gamré* and *Soudamouka* to contribute meaningfully to dietary quality among infants and young children in a region where undernutrition remains widespread. As highlighted earlier, more than 40% of children under five are stunted, and deficiencies in iron, zinc, and vitamin A remain widespread (INS and ICF 2020; Ministry of Public Health 2009). Identifying nutrient‐dense, culturally acceptable, and locally available foods is therefore essential for strengthening complementary feeding practices and addressing persistent micronutrient gaps.

### 4.1 Nutrient Density of Small Indigenous Fish

The nutrient composition results confirm that dried small fish are highly concentrated sources of protein, fat, calcium, iron, zinc, and vitamin A. The substantial increase in nutrient density following drying aligns with previous work showing that moisture reduction concentrates macro‐ and micronutrients in small fish species (Bogard et al. 2015; Rasul et al. 2022). In this study, dried samples contained up to 64 g protein/100 g and more than 1400 mg calcium/100 g in some species—values comparable to those reported in Bangladesh and India (Bogard et al. 2015; Pushp et al. 2024). These findings reinforce the well‐established role of small fish as affordable sources of bioavailable micronutrients, particularly when consumed whole (Kawarazuka and Béné 2011; Thilsted et al. 2016).

The contribution analysis further demonstrates the practical relevance of these foods for young children. A 10 g portion of dried small fish provided 40%–50% of the daily protein requirement from complementary foods and up to one‐third of the calcium needs for children aged 6–21 months. Although iron contributions were modest relative to physiological requirements, they remain nutritionally meaningful in settings where few animal‐source foods are consumed (Black et al. 2013). These results support global recommendations emphasizing the inclusion of nutrient‐dense animal‐source foods in complementary feeding (Dewey 2003; WHO/FAO 2004).

### 4.2 Household Knowledge, Practices, and the Awareness‐Use Gap

Despite high awareness (89.4%) and widespread consumption (92.5%) of small fish, only 24.6% of households reported using them to enrich children's meals. This gap between availability, acceptability, and actual use is a critical finding. Several underlying factors may explain this paradox:

- Knowledge deficits: although small fish are culturally accepted and widely consumed by adults, their nutritional value for young children is not fully leveraged. Limited knowledge appears to be a key contributing factor, as 23.5% of caregivers reported not knowing which foods were appropriate for enrichment, indicating limited guidance on how to safely and effectively incorporate small fish into complementary feeding. Similar gaps have been documented in other low‐income settings, where caregivers often lack practical instruction on integrating nutrient‐dense foods into child diets (Agyei‐Mensah et al. 2023; Black et al. 2013).
- Perceived appropriateness: caregivers may perceive small fish as suitable for adults and older children but less appropriate for infants, reflecting cultural feeding norms. Research from Asia and Africa indicates that cultural perceptions regarding the age suitability of fish, along with concerns about choking or digestive difficulties, frequently delay its introduction during complementary feeding (Thilsted et al. 2016; Yigit et al. 2025).
- Practical barriers: preparation challenges such as removing bones, achieving suitable texture, and concerns about hygiene may discourage caregivers from offering small fish to young children. Evidence from complementary feeding studies indicates that fear of fishbones and preparation difficulties are among the most cited barriers to fish use in infant diets (Bogard et al. 2015; Yigit et al. 2025).
- Information asymmetry: while caregivers recognize the taste and general nutritional value of small fish, specific knowledge of their micronutrient contributions (e.g., calcium, iron, zinc, vitamin A, and essential fatty acids) is often lacking. Research shows that families receiving nutrition counseling are significantly more likely to introduce fish early and frequently, highlighting the role of targeted information in bridging this gap (Bavinck et al. 2023).

The very high willingness to include small fish in children's diets (91.8%) indicates that cultural resistance is not a barrier. Instead, the findings point to a knowledge‐practice disconnect, highlighting the need for targeted nutrition education, demonstration of preparation techniques, and behavior‐change communication to translate awareness into consistent child‐focused use.

Multivariate analysis further emphasizes the role of education, urban residence, and household head occupation in shaping complementary feeding practices. Traders and fishers may have greater exposure and access to small fish, facilitating their use in children's meals. Household size and religion did not retain significance after adjustment, suggesting that their influence may be mediated by socioeconomic context. Targeted interventions should therefore prioritize caregiver education, rural access, and occupational engagement in fish value chains to enhance utilization.

### 4.3 Acceptability of Fish Powder and Implications for Adoption

The standardized fish powder was well accepted, with favorable sensory ratings for taste, odor, and overall appreciation. Caregivers consistently reported ease of incorporation into common child foods, suggesting strong feasibility for household adoption. These findings are consistent with evidence from similar studies in Asia and Africa, where sensory acceptance has been identified as a critical determinant of successful complementary feeding interventions (Thilsted et al. 2016; Pushp et al. 2024).

Importantly, the study population was characterized by high levels of acute and chronic malnutrition, with one‐third of children classified as wasted and more than one‐third underweight. In such nutritionally vulnerable groups, positive sensory acceptance highlights the potential of small fish powders as a culturally appropriate, nutrient dense food‐based strategy. Similar studies in Bangladesh and India have demonstrated that small fish powders can be feasibly integrated into household diets and contribute meaningfully to protein and calcium adequacy (Bogard et al. 2015; Pushp et al. 2024).

Sensory evaluation revealed heterogeneity across attributes. High ratings for taste and overall appreciation suggest strong alignment with consumer expectations, facilitating integration into household diets. However, comparatively lower perceptions of appearance and texture highlight potential barriers to sustained use, particularly in infant feeding contexts where caregivers are attentive to consistency and presentation.

Evidence from complementary feeding interventions in other settings confirms that caregiver acceptance is decisive for adoption. In Zambia, sensory panel studies of dishes fortified with dried fish powder (ComFA+Fish) demonstrated high caregiver and infant acceptability across multiple attributes, including taste, aroma, and ease of preparation (Ragsdale et al. 2024). In Malawi, complementary feeding trials showed that small fish powder improved infant growth and micronutrient status, with caregivers reporting strong acceptance despite occasional concerns about texture (Ahern et al. 2025). Broader analyses reinforce that sustaining healthy diets requires integration of nutrient‐rich small fish into both capture fisheries and aquaculture systems, with sensory acceptability remaining a critical determinant of uptake (Thilsted et al. 2016).

From a programmatic perspective, the favorable hedonic profile supports the feasibility of introducing small fish powders into infant and young child feeding (IYCF) strategies. To translate initial willingness into sustained practice, product development should prioritize improvements in texture and appearance through optimized processing and packaging. Equally important is caregiver education, which can build confidence in preparation methods and highlight nutritional benefits. Integration into IYCF counseling and community‐based nutrition programs would help bridge the awareness–use gap and strengthen adoption at scale.

Taken together, the findings suggest that small fish powders are not only nutritionally valuable but also culturally and sensorially acceptable. To translate this potential into sustained practice, interventions should combine product development with nutrition education, caregiver training on safe preparation methods, and integration into IYCF counseling programs. Such strategies would help bridge the gap between high willingness to adopt and consistent use, positioning small indigenous fish as a scalable solution for improving complementary feeding in northern Cameroon.

### 4.4 Food Safety Consideration

In addition to nutritional potential, the safety of fish powder intended for infant complementary feeding is a critical consideration. Fish lipids, particularly polyunsaturated fatty acids, are highly susceptible to oxidative degradation, which can compromise both quality and palatability if storage conditions are inadequate (Sikorski and Kolakowska 2002). Moreover, microbial contamination and pathogenic microorganisms pose significant risks, especially for infants who are more vulnerable to foodborne illness. Controlled cooking, hygienic drying, and milling under sanitized conditions, combined with packaging in airtight food‐grade containers, were applied to minimize these hazards (FAO/WHO 2009).

Although aflatoxins are primarily associated with cereals and legumes, cross‐contamination during storage remains a possible hazard when fish powder is kept alongside contaminated products. Preventive measures such as segregated storage, moisture control, and routine monitoring are therefore essential (Wild and Gong 2010). To enhance product safety, implementing Hazard Analysis and Critical Control Point (HACCP) systems is advised, as they provide a structured framework for systematically identifying, monitoring, and controlling risks throughout the production chain (Mortimore and Wallace 2013).

Future research should integrate comprehensive safety assessments, including lipid stability, microbial load, and aflatoxin screening, to ensure that fish powder can be positioned not only as a nutritionally beneficial but also a safe and reliable complementary food ingredient for infants.

### 4.5 Study Limitations and Future Research

Several limitations should be acknowledged. First, the study employed an observational design, which restricts causal inference regarding the impact of small fish consumption on child nutrition outcomes. Observational approaches are valuable for generating context‐specific evidence but cannot establish direct cause–effect relationships (Black et al. 2013).

Second, household survey data relied on caregiver self‐report, which may be subject to recall and social desirability bias. Similar challenges have been documented in infant and young child feeding (IYCF) research, where caregivers often over‐report desirable practices or under‐report feeding difficulties (Gatica‐Domínguez et al. 2021; Agyei‐Mensah et al. 2023).

Third, the acceptability assessment was conducted under controlled conditions using standardized fish powder. While this provides valuable insights into immediate sensory responses, it may not fully reflect household practices, variability in preparation methods, or long‐term adherence. Studies in other settings have shown that although fish powders are acceptable in short‐term trials, sustained use depends on household convenience, affordability, and integration into daily routines (Pushp et al. 2024).

Fourth, the study was limited to selected localities in northern Cameroon. Although these sites were purposively chosen for their relevance to local dietary practices, findings may not be generalizable to other regions with different cultural norms, food environments, or socioeconomic conditions. This limitation is common in community‐based nutrition studies, where contextual specificity strengthens local relevance but constrains external validity (Thilsted et al. 2016).

Finally, the study did not include fatty acid or amino acid profiling, which would have enriched the nutritional characterization by providing insights into lipid and protein quality. These analyses were not conducted due to resource constraints, but future research should incorporate them to strengthen the evidence base.

Future studies should therefore:

- Apply longitudinal or intervention designs to assess causal effects of small fish consumption on child growth and micronutrient status.
- Incorporate multivariate analyses to identify determinants of household utilization, including socioeconomic, educational, and cultural factors.
- Evaluate product stability and safety under household storage conditions, including risks of lipid oxidation, microbial contamination, and aflatoxin exposure.
- Conduct detailed fatty acid and amino acid profiling to clarify the nutritional quality of small indigenous fish.
- Explore scalable community‐based production models and culturally tailored behavior‐change strategies to enhance caregiver adoption and sustained use.

Together, these directions will help position small indigenous fish as a robust, locally available, and sustainable food‐based solution to improve child nutrition in resource‐constrained settings.

## 5 Conclusion

This study shows that small indigenous fish species—particularly *Gamré* and *Soudamouka*—are nutrient‐dense, culturally accepted, and locally accessible foods with potential to enhance complementary feeding in northern Cameroon. Their dried forms provide substantial amounts of protein, calcium, iron, zinc, and vitamin A, indicating that even small portions could contribute meaningfully to the dietary adequacy of young children.

The novelty of this work lies in its integrative design, combining nutrient composition analyses with household surveys and acceptability testing. This approach links biological evidence with cultural practices and caregiver perceptions, highlighting both the nutritional potential of small fish and the gap between high household awareness and their limited use in child feeding.

At the same time, the conclusions must be interpreted with caution. The study was observational, and while the association between small fish consumption, nutrient contributions, and acceptability were demonstrated, causal impacts on child nutrition outcomes cannot be inferred. Household practices were self‐reported, which may introduce recall or social desirability bias, and the acceptability assessment was short‐term and conducted under observation, potentially influencing caregiver responses. These limitations highlight the need for further longitudinal and intervention studies to confirm sustained adoption and nutritional impact.

Despite these constraints, the findings highlight important opportunities for food‐based strategies. Addressing the awareness‐use gap through nutrition education, caregiver training on safe preparation methods, and improved household level processing could support more consistent use of small fish in children's diets. Strengthening local value chains and integrating fish powders into infant and young child feeding counseling may further enhance feasibility.

Future research should therefore examine the long‐term effects of regular small fish consumption on child growth and micronutrient status, evaluate the stability and safety of fish powders under typical household storage conditions, and explore scalable models for community‐based production. By pursuing these directions, small indigenous fish can be more rigorously positioned as part of integrated, evidence‐based approaches to improving child nutrition in northern Cameroon and similar contexts.

## Author Contributions

**Abomo Ndzana Anne Christine:** data curation, writing – original draft, methodology, formal analysis, visualization. **Essa'a Veronique Josette:** writing – review and editing, investigation, visualization. **Medoua Nama Gabriel:** conceptualization, methodology, investigation, formal analysis, data curation, project administration, supervision, writing – review and editing. **Socpa Antoine:** formal analysis, writing – review and editing, supervision. **Nankap Martin:** project administration, funding acquisition, writing – review and editing.

## Funding

This study was financially supported by UNICEF—Cameroon.

## Disclosure

All authors have read and approved the final version of the manuscript. Corresponding Author had full access to all of the data in this study and takes complete responsibility for the integrity of the data and the accuracy of the data analysis.

## Ethics Statement

This study was approved by the Ministry of Public Health Review Board of North Regional Ethics Committee for Human Health Research.

## Consent

The authors have nothing to report.

## Conflicts of Interest

The authors declare no conflicts of interest.

## Data Availability

The data that support the findings of this study are available from the corresponding author upon reasonable request.
